# Lecture on Pneumonia,—Jaundice,—Tubercular Meningitis,—Pathology of Laryngitis and Laryngeal Phthisis

**Published:** 1840-02-08

**Authors:** W. W. Gerhard


### CLINICAL LECTURE.

### PHILADELPHIA HOSPITAL.

*Wednesday, January 22d and 29th,* 1840.

**LECTURE ON PNEUMONIA,—JAUNDICE,—TUBERCULAR MENINGITIS,—PATHOLOGY OF LARYNGITIS AND LARYNGEAL PHTHISIS.**

By W. W. Gerhard, M. D.

*Nos.* 10 *and* 11—*Winter Course.*

I shall exhibit to-day several patients convalescent from acute diseases, especially inflammatory affections of the lungs. I shall conclude the lecture with a few remarks on the tubercular affection of the membranes of the brain, so common among children, and with the demonstration of some pathological specimens illustrating the history of laryngitis.

*Case* 1.—In the two preceding lectures I spoke to you of a man labouring under an acute disease of the lungs, the severity of which rendered it improper to remove him from the ward. He is now before you in a state of convalescence. This man entered the hospital about a month since, at which time he presented the signs of pneumonia, complicated with an incipient tubercular affection—the latter impressing certain modifications upon the characters of the former. Under the treatment which was adopted, the patient at first did very well; but suddenly grew much worse, in consequence of the supervention of pleurisy, followed by copious effusion. The distress produced by this complication added to that immediately consequent upon the proper affection of the lungs, and brought the patient to the brink of the grave. The dyspnœa and prostration of the system were extreme. In this condition of things, a mild, depletive plan, was pursued; the great depression of the recuperative powers of the patient put all active measures of the kind out of the question. A single cup was, therefore, applied, and repeated according to the necessity of the case; at the same time the patient was put upon the use of mercury.

This treatment was soon followed by an improvement in the aspect of the case, which, however, was not very decided, until the action of the mercury was made manifest by the occurrence of slight ptyalism. From that time, (seven or eight days since,) the patient has been steadily improving. But convalescence, in a case like the present, is never so rapid as it is in simple pneumonia. This is owing partly to the tubercular diathesis of the patient; in part, also, to the abundance of the pleuritic effusion, which always requires a certain time for its removal by absorption, more particularly when it co-exists with pneumonia or tubercles. The case is one of considerable interest, as illustrating the advantages of very moderate depletion, and of the judicious employment of mercury in certain forms of acute pulmonary disease. In relation to the latter remedy, I have repeatedly urged it upon your attention, that we employ it in acute affections, not for the purpose of salivating our patients, but of producing its specific constitutional influence. Its effect upon the mouth is the mark of this influence, or of the saturation of the system with mercury: after the slightest possible redness of the gums is produced, the influence of the remedy cannot be rendered more complete and decided, no matter how copiously it be given.

*Case* 2.—This is an instance of pneumonia so mild as hardly to require any treatment. The patient is a man aged forty-nine; he is of robust frame, and has led an active life, having been for some years a soldier in the Peninsula; he has generally enjoyed good health. He has lately been employed in cutting ice. On the 19th he was taken ill; the first sign of the disorder was a chill. This was followed by cough, but no pain in the chest; the expectoration has been viscid from the first, but has never been coloured with blood; at no time has the patient been confined to bed. On the 25th he entered the hospital,—and, on examining his chest, the physical signs of pneumonia were detected. The following pectoral mixture was ordered:

| | | |
|---|---|---|
| ℞. | Ext. Hyoscyami, | gr. viij. |
| | Syrup Polyg. Senegæ, | ℥j. |
| | Mucilag. Acaciæ, | ℥v. |
| Ft. | Mist. | |

*27th.*—The hot infusion of eupatorium was ordered. Under the action of this remedy, the disease passed off by a copious diaphoresis: the patient is still sweating. He may now be considered as convalescent. It would appear, therefore, that only eight days elapsed from the commencement, to the crisis of the disease. This seems to be in contradiction to a rule which I laid down in one of the preceding lectures, viz.: that the usual duration of pneumonia is from fourteen to twenty-one days. But it is to be remarked, that, although the general signs indicate the declension of the disease, the local signs still exist; there are yet manifest, some dulness on percussion, and crepitant rhonchus. By the time that these have disappeared, the case will probably be brought within the average which I have stated. This is the usual course of mild cases of pneumonia.

In cases like the present, no active treatment, and little, indeed, of any kind is requisite. Hot diaphoretic and nauseating drinks, are the best possible remedies. Such instances of mild disease are less frequent in pneumonia, than in most other diseases; the expectant and palliative treatment just mentioned, is rarely worthy of confidence. But when such cases do occur, a more active treatment only puts the patient to inconvenience, without resulting in adequate benefit. I wish particularly to inculcate this principle, that, when a disease is tending to a favourable termination, all measures of an active kind should be dispensed with; it should be a matter of conscience with the physician not to harrass the patient with unnecessary applications. An opposite course of practice tends, in no small degree, to throw discredit on the profession. This hurtful officiousness arises not from any defect or error in the plan of instruction in our schools, but from an idea which is so apt to be entertained by every student from the commencement of his studies, viz.: that every disease is recognised by certain signs, and requires for its cure, a certain set of remedies. So that whenever the symptoms indicate the existence of inflammation, bleeding, purgation, and revulsion at once suggest themselves to his mind. It requires experience to teach him that there are, in fact, many cases of inflammation, and still more of continued fevers, which require no such violent measures.

*Case* 3.—In this case we have pursued a plan of treatment precisely the reverse of that adopted in the preceding one. Very free bleeding, and other depletory measures have been resorted to, but not with a corresponding amelioration of the symptoms, because they were not practised until the disease had made considerable progress. The patient, Shepherd, was seized with pneumonia on the 8th inst.; he was admitted into the hospital on the 18th; so that ten days had elapsed before any attack upon the disease was made. The treatment was commenced by the abstraction of twenty ounces of blood from the arm; this was followed by cups to the side, which were repeated three times; the patient was also placed upon the use of the infus. eupatorii. Notwithstanding these active measures, the disease continued to advance, with very slight modification of the symptoms. The combination of opium, digitalis, and calomel, of which I have already so often spoken, was then ordered, mainly with the view of obtaining the antiphlogistic action of the mercury. Two days after his entrance, the patient was taken with singultus, which came on at particular periods in the day, and continued for an hour or two at each attack. Hiccough is a symptom of grave import in inflammatory affections, and is injurious in itself, inasmuch as it serves to exhaust the strength of the patient. It seems not to depend directly on the inflammation, but on a sympathetic irritation of the nerves of the diaphragm.

It is a symptom which is more frequently met with in some seasons than in others; during the past year, we have not had many instances of it. As it was not quieted by the treatment already adopted, assafœtida was ordered with this view; this failing, the oil of amber was ordered, in doses of six drops, repeated according to circumstances. Under this treatment, the singultus gradually subsided, and the disease took a favourable turn.

At the last lecture I remarked to you that the duration of this case would probably not be shortened by the active practice which had been pursued, and the result has verified the remark. Bleeding, however copious, will not cut short an inflammatory disease, unless practised soon after its invasion; a few hours may carry the affection beyond the point at which depletion may cause it to abort. After it has passed this point, bleeding, though it does not arrest the course of the disease, is still of use, by palliating its inconveniences, and diminishing its tendency to run into fatal disorganizations.

Little change was perceptible in the condition of the patient until the 25th, when he was somewhat better. On the 26th he appeared to be in a state of convalescence. On the 27th this favourable change was still more evident. The face was pale and sunken; I have already stated that this subsidence of the features after the fulness and flushing produced by inflammatory excitement, is one of the best signs of convalescence. The pulse had fallen from ninety-six to eighty; it was soft and tremulous. The respiration had also fallen from thirty to twenty in the minute. No doubt therefore could exist as to the fact of the patient's convalescence; the simultaneous subsidence of the respiration and the pulse rendered it perfectly certain. On the contrary, if the pulse had become slower, while the respiration retained its frequency, we should have concluded that the patient was in a much worse condition.

The duration of this attack of pneumonia was nineteen days, which is within the average which I stated in my remarks upon case 1. Pneumonia, in fact, has a natural duration, and one principal object to be aimed at in its treatment, after the disease is established, is to prevent accidental circumstances from interfering with the natural tendency of the disease to terminate at a certain period.

*Case* 4.—This man, aged 45, entered the hospital on the 23d inst. He had always enjoyed good health until last summer, when he had an attack of dysentery. On the 22d, after exposure to wet and cold, he was seized with cough, and the same evening he had a chill, attended with pain at the xiphoid cartilage. On the 23d he was jaundiced; the skin, conjunctiva, and urine, were of a deep yellow, and every object appeared to his sight to be of the same colour; the skin was moderately warm; there was no headach, but pain and tenderness in the right hypochondrium, which obliged the patient to lie on the left side; expectoration was slight; pulse moderately full. Venesection, cupping over the region of the liver, and a diaphoretic infusion, were ordered.

*24th.*—Patient more jaundiced; pain had extended to epigastrium; slight signs of bronchitis observed. Cups repeated, and an infusion of senna, with sulphate of magnesia, ordered.

*26th.*—Patient now complained of headach and vertigo, depresssion of mind, &c. Bleeding repeated.

From this time the symptoms rapidly abated, and few remains of the disorder are now perceptible. The skin is only slightly coloured on the breast; the vision has become natural; the skin is moist; it has never been hot, however, at any time in the progress of the case.

The most prominent symptom of this case, besides the alteration in the colour of the skin, was the tenderness in the right hypocondriac and epigastric regions, accompanied by dulness on percussion. It was inferred from these signs that the liver was congested, and slightly inflamed. Inflammation of the liver is by no means a common occurrence in the winter season. The prevailing inflammatory diseases are those of the lungs, heart, and fibrous tissues of the extremities; the abdominal viscera are more rarely affected. But this man's previous history affords a very sufficient reason for the occurrence of hæpatitis in his case. Last summer he had an attack of dysentery; now, a severe dysentery hardly ever passes through its course without involving the liver in a greater or less degree. There is, therefore, a strong presumption, that this man's liver was at that time affected; it was naturally left in a condition favourable to the return of disease, upon the occurrence of the usual causes. This circumstance determined the deleterious impression of cold to the liver rather than to the lungs.

Jaundice depends on a great variety of causes. When acute, as in the present case, and dependent on congestion and slight inflammation of the liver, it is in general easily cured. It yielded in this case to bleeding, cupping over the liver, and saline purgatives. Mercury was not employed at all in the treatment; the only character in which it could have been used with propriety, was that of an evacuant,—and in this case it seemed to offer no particular advantages over saline and other purgatives. You will recollect that in the numerous instances recently brought to your notice in which this article was employed, it was not as a purgative, but as an antiphlogistic remedy in certain stages of inflammatory affections.

Headach was an important symptom in this case, and its occurrence induced us to repeat the bleeding. In all cases of jaundice, indeed, cerebral symptoms demand particular attention; for it is usually in consequence of the supervention of cerebral affections, that this disease proves fatal. The cause of this complication is the suppression of the biliary secretion, the elements of which being retained in the blood, act like a poison upon the system, especially on the brain. In like manner, urea, if retained in the blood, proves deleterious. In fact, all diseases of the liver or kidneys, attended with suppression of their secretions, are followed by coma, and other signs of cerebral oppression, in consequence of which they terminate fatally. It is in this way that the granular affection of the kidneys, called "Bright's disease," often proves fatal. When the cerebral symptoms are active, the proper treatment is general and local bleeding, cold applications, &c. But it is impossible to remove them until the bile is eliminated from the blood; this is effected slowly, and by a process of nature. If the symptoms are attended with much depression of the vital energies, depletion becomes improper, and we have to rely on other means, the most effectual of which experience proves to be sinapisms and blisters.

At the last lecture I introduced a man labouring under acute laryngitis, upon whom an operation was performed in your presence, but without success. This case was one of acute, grafted on chronic, laryngitis. The affection was originally acute, (having commenced about a year ago,) but became chronic, and continued so till ten days before the patient's entrance; it then became acute, and the symptoms were strongly marked at the time of his entrance. It was likewise observed that the lungs were affected,—but in what way, the signs were too obscure to enable us precisely to determine. The lungs were pervious to the air, so that there was little dulness on percussion; but the respiration was feeble throughout the right side, and some crepitus was distinguished below. It was hence inferred that the lung was congested, but nothing else could be made out with any certainty: as you will presently see, these signs were owing to the development of miliary, tubercular granulations, in great abundance, with congestion of the surrounding tissues. The operation of laryngotomy, performed by Dr. Gibson, produced some relief for the moment; but the dyspnœa returned every time the artificial opening became obstructed, and not more than half an hour elapsed before the man died of suffocation. The operation was resorted to as the only chance of prolonging life; but even if it had been more successful for the time, it could not, in the end, have saved the patient—for an immense number of grayish, semi-transparent, tubercular granulations, had already been deposited in all the lobes of the right lung, and in the upper, and a part of the lower lobe of the left. But, had the condition of the patient been such as to allow a full examination and positive diagnosis, the operation would still have been justifiable, as the only means of securing to the patient a few more hours or days of life.

The immediate cause of the intense dyspnœa under which the patient laboured, was œdema of the larynx, and inflammation of the trachea and bronchiæ. The former offered a very great obstacle to the passage of the air through the rima glottidis,—while the trachea and bronchi were lined by a layer of very viscid mucus, which interposed a further obstacle to its entrance into the lungs. The matter lining the air-passages was not, properly speaking, a false membrane, but it was so dense that it could be detached in shreds of considerable length. There was likewise an ulceration of the lining membrane of the larynx, between the posterior extremities of the vocal cords, and extending to the cricoid cartilage.

This case is one of interest, inasmuch as it illustrates the connexion between laryngitis and phthisis. Laryngitis sometimes occurs as the primary, sometimes as the secondary disease. The latter was the case in a patient who lately died in the female wards; during the progress of a tubercular affection, she was attacked with laryngitis, which was indicated by the ordinary symptoms, dyspnœa and aphonia. When laryngitis occurs as the original affection, it may continue for years, attended with more or less cough, hoarseness, and dyspnœa, but without any indications of disease in the pulmonary tissue. At last ulceration occurs; at this point, in a very large proportion of cases, tubercles are developed in the lungs. In this variety of phthisis the tubercles are generally grayish, semi-transparent granules, of small size, and uniformly diffused through the lung. Consequently, phthisis following laryngitis, is one of the most intractable varieties of the disease. In all cases where the affection of the larynx has advanced to ulceration, we apprehend the supervention of phthisis. Almost the only variety forming an exception to the rule, is that form of ulceration of cartilages and of the mucous membrane which occurs in secondary syphilis. But it is easy to discriminate such cases by the general condition of the patient, the history of the affection, &c. Besides, ulceration of the larynx, consequent on syphilis, almost always extends rapidly into the cartilages. The prognosis of laryngitis is never grave until ulceration has occurred: if œdema is the only termination, a cure may frequently be effected; but if ulceration takes place, this result can hardly be hoped for.

If the dyspnœa should be excessive, and threaten suffocation, an operation for its relief is the only resource. There are several different methods of performing such an operation. Dr. Gibson, in the case which we have been considering, preferred laryngotomy. This is usually done by making a transverse incision through the crico-thyroid membrane. In France, the operation for croup is often performed, and at the present day surgeons generally are in favour of tracheotomy. In this operation, a longitudinal incision is made into the trachea, which is kept open for the passage of air, either by means of a canula, or of a blunt hook applied to each edge of the incision. This operation is preferred to laryngotomy; inasmuch as it admits of a more extensive opening, through which the false membrane may be pulled away. You will find an excellent paper on this subject in a late number of the Medical Examiner.

I now show you the larynx and trachea of the man who died after the operation of laryngotomy was performed. The lining membrane of the larynx was at first highly injected; but, by maceration in water, the blood has been almost entirely washed out. However, you can still perceive the œdematous state of the glottis. This œdema was produced by the effusion of serum under the mucous membrane, in the same manner that it is effused from the surface of inflamed serous membranes. The mucous membrane is softened: at the posterior part of the larynx is a large ulcer, and many smaller ones are scattered over the remaining portions of the larynx, as well as the upper part of the trachea. The mucous membrane of the trachea, like that of the larynx, was highly injected. The epiglottis is slightly thickened at its lower part. In the crico-thyroid membrane, you see the opening made by the operation; it is not quite so large, in fact, as the natural opening of the glottis; it therefore easily became obstructed by the viscid secretions which filled up the air-passages.

The upper lobe of the left lung, and all the lobes of the right one, contain an immense number of small, gray, semi-transparent granulations. In the right lung, they are so numerous as to have almost obliterated its vesicular structure. A tubercular deposition of this kind never occurs, except in acute phthisis. The right lung is likewise congested, particularly at the lower part, over which the crepitant rhonchus was heard during life. At the summit of the lung is a cicatrized cavity, containing a mass of calcareous matter. The surrounding parenchyma is puckered and contracted by the cicatrix, and the adjacent pleura is covered by adhesions. These appearances indicate the former existence of tubercles; these were probably deposited at the same time the chronic laryngitis occurred, and were removed by the absorption of their animal matter, the calcareous portion remaining behind, and constituting the white masses which you here see.

Here are the lungs of another patient, who entered the hospital in the last stage of phthisis, and died within forty-eight hours. I show them to you for the purpose of contrasting the early, with the latter stages of the affection. At the summit of one lung is a large cavity, and in that of the opposite one, are numerous small granulations, of recent origin.

When the tubercular granulations are deposited in great numbers through the pulmonary tissue, the disease is almost always acute, and, in fact, is identical with what is often called the "galloping consumption." The termination of this variety, which is almost always fatal, occurs in two ways; in the one the patient dies of dyspnœa, and you find, as in the present case, the lungs excessively congested, through all that portion of them in which the granulations are deposited. The death then actually takes place by suffocation. I have seen some examples of it; one of the first was twelve years ago, when I was a resident pupil of this hospital. A black, who had been labouring under the disease for some time, with much dyspnœa, called to us one day while making the visit, that he was strangling, and died almost immediately; the lungs were excessively congested, and almost stuffed with these granulations.

In most cases, the disease passes on to softening at the summit, at least of the lungs, while the rest of the tissue is engorged and filled with the granulations. In these cases there is high fever, sweating, and generally intense dyspnœa. The cough, however, may be very slight. You will find the respiration generally feeble, and the chest less sonorous than usual.

There lately occurred a disease of the brain, which I am accidentally prevented from showing you, and which was interesting as an illustration of an affection of which I may perhaps speak more fully at a future time,—tubercular disease of the membranes of the brain. The case was that of an adult. On opening the cranium, tubercular granulations were found beneath the membranes, both on the superior surface and base of the brain; in the intervals left by them, the membranes were injected, and covered with lymph. The affection probably followed the development of tubercles in the lungs; the examination was not extended to these organs. The deposition of tubercles under the membranes of the brain was followed by acute inflammation, which resulted in effusion, and softening of the cerebral substance. This was indicated by rigidity and paralysis of the extremities; muttering delirium; subsultus tendinum; contraction, and afterwards dilatation of the pupils; distortion of the mouth. The occurrence of symptoms of meningitis in the course of tubercular phthisis, may be considered sufficiently certain evidence of the development of the affection of which I am speaking. In children it is indicated by the signs commonly described as belonging to acute hydrocephalus. This disease so called, which is frequent from the age of two years up to puberty, is neither more nor less than tubercular meningitis ; the inflammation is usually attended with effusion into the ventricles or on the surface of the brain, and from this circumstance, the ordinary appellation of the disease is derived. But the effusion is altogether an accidental matter; and so is the softening which sometimes occurs. It is the tubercular deposite, and the concomitant inflammation, which constitute the essential characteristics. It is only of late years, that the true nature of this affection was pointed out. In 1832 or 33, the first connected observations on the subject were made by Dr. Rufz and myself, during a residence in Paris, and attendance at the Hôpital des Enfans. Of late, it has attracted much attention in France, and the truth of the observations concerning it, is now admitted. In our country, it is as yet, scarcely attended to, by most pathologists, and so that even Dr. Gross, in his late excellent work on Pathological Anatomy, passes the subject over almost without a notice. In concluding my remarks upon it for the present, it is proper to observe, that, although sometimes curable in children, tubercular meningitis, in most cases, proves rebellious to treatment.

[The press of matter obliges us to omit the greater part of one of these lectures, on the therapeutic effects of mercury in inflammatory diseases, &c. It is well to state to our readers that these lectures are carefully noted as delivered, by a member of the class. They are revised by the lecturer, and such alterations made as are rendered necessary to insure correctness. This will account for some peculiarities in expression, &c.]

